# Ru(II) Complex Grafted Ti_3_C_2_T_x_ MXene Nano Sheet with Photothermal/Photodynamic Synergistic Antibacterial Activity

**DOI:** 10.3390/nano13060958

**Published:** 2023-03-07

**Authors:** Xiaofang Liu, Hongchi Xie, Shi Zhuo, Yuanhong Zhou, Mohamed S. Selim, Xiang Chen, Zhifeng Hao

**Affiliations:** 1 Key Laboratory of Clean Chemistry Technology of Guangdong Regular Higher Education Institutions, School of Chemical Engineering and Light Industry, Guangdong University of Technology, Guangzhou 510006, China; 2 Egyptian Petroleum Research Institute, Petroleum Application Department, Cairo 11727, Egypt

**Keywords:** MXene, Ru complex, PTT/PDT, antimicrobial

## Abstract

For a long time, the emergence of microbial drug resistance due to the abuse of antibiotics has greatly reduced the therapeutic effect of many existing antibiotics. This makes the development of new antimicrobial materials urgent. Light-assisted antimicrobial therapy is an alternative to antibiotic therapy due to its high antimicrobial efficiency and non-resistance. Here, we develop a nanocomposite material (Ru@MXene) which is based on Ru(bpy)(dcb)^2+^ connected to MXene nanosheets by ester bonding as a photothermal/photodynamic synergistic antibacterial material. The obtained Ru@MXene nanocomposites exhibit a strengthened antimicrobial capacity compared to Ru or MXene alone, which can be attributed to the higher reactive oxygen species (ROS) yield and the thermal effect. Once exposed to a xenon lamp, Ru@MXene promptly achieved almost 100% bactericidal activity against *Escherichia coli* (200 μg/mL) and *Staphylococcus aureus* (100 μg/mL). This is ascribed to its synergistic photothermal therapy (PTT) and photodynamic therapy (PDT) capabilities. Consequently, the innovative Ru@MXene can be a prospective non-drug antimicrobial therapy that avoids antibiotic resistance in practice. Notably, this high-efficiency PTT/PDT synergistic antimicrobial material by bonding Ru complexes to MXene is the first such reported model. However, the toxic effects of Ru@MXene materials need to be studied to evaluate them for further medical applications.

## 1. Introduction

The COVID-19 pandemic pushed public health to a threshold of collapse in 2020. As the COVID-19 pandemic drives up the usage of antibiotics, it will eventually contribute to higher rates of bacterial resistance and affect the disease burden and mortality rates [1]. The emergence and widespread spread of superbugs has become a challenging health issue worldwide owing to the dramatic evolution of drug-resistant genes in bacteria [2,3,4,5]. Accordingly, it is imperative that new, reliable and effective antimicrobial approaches are available globally to combat the escalation of bacterial resistance. Compared to traditional antibiotic sterilization, light-controlled sterilization not only cures the infection and eliminates inflammation effectively in a short period of time, but it also has the advantage of less toxicity and fewer side effects [6,7,8]. Thus, numerous researchers have devoted themselves to the study of light-triggered sterilization strategies, such as activated oxygen species (ROS)-associated photodynamic therapy (PDT) and thermo-induced photothermal therapy (PTT), both of which strongly rely on the synthesis of light-activated nanomaterials [9,10,11,12].

Currently, the most well-known categories of photothermal materials include precious metal nanoparticles [13,14], metal oxides [15], metal sulphides [16,17], carbon materials [18,19,20,21,22,23], phosphorus monomers [24] and polymers [25,26]. Taking advantage of the functional nanomaterials, it is feasible to integrate PDT and PTT into a monolithic platform where synergistic treatment can remarkably enhance the antimicrobial capacity. To illustrate, Wang and his coworkers reported an urchin-shaped Au@Bi_2_S_3_ core-shell nanoparticle with a synergistic NIR-triggered function of photothermal/photodynamic effects for bacterial sterilization [27]. However, two–dimensional nanomaterials of transition metal carbides (MXenes) have gained increased attention recently for biological applications, as they are characterized by a large specific surface area, high electrical conductivity [28], outstanding biocompatibility [29,30], excellent photothermal conversion [31] and ease of functionalization [6,32]. Therefore, it can be used as a photo-induced antibacterial agent. The abundance of functional groups (-OH, =O and -F) on Ti_3_C_2_ MXene’s surface gives it anchor points. Reports have already been published on the photothermal antimicrobial therapy application of MXene combined with other materials. For instance, Liu et al. [33] developed a heterogeneous structured coating (MXene/CoNWs) that achieves a greater than 90% antibacterial rate against Gram-positive/Gram-negative bacteria within 20 min due to the synergistic effect of NIR-triggered ROS and thermal therapy. However, visible light-assisted therapy is more accessible and available than NIR light. Cheng et al. [34] constructed organic–inorganic hybrids of ZnTCPP/Ti_3_C_2_T_X_ by a hydrothermal method, which enhanced visible light absorption and catalysis, while ZnTCPP/Ti_3_C_2_T_X_ could trap bacteria electrostatically and significantly improved visible light sterilization efficiency. These active antibacterial nanomaterials have the potential to be made available for diverse practical applications, such as medical devices, textiles and building materials, where its antimicrobial properties could help prevent the growth and spread of hazardous bacteria. As an illustration, active antibacterial nanomaterials can be coated on the surface of medical masks to eliminate bacteria or viruses against cross-infection; in addition, they can also be applied to prepare antibacterial walls, antibacterial flooring and antibacterial windows and doors, which may effectively kill bacteria and contribute to better indoor hygiene.

Heretofore, light-responsive antibacterial properties of MXene covalently bonded with ruthenium complexes and coupling PTT and PDT properties by xenon lamp has barely been reported. Among the transition metal series, ruthenium can form very strong ruthenium(II) complexes due to its lower energy barrier (0.125 nm), easy solubility in water, small atomic size, and high nuclear charge [35]. The centrally located ruthenium atom is actually a structural center that supports a three-dimensional ligand skeleton with a rigid structure, allowing the ligand to be easily substituted or modified to generate numerous ruthenium(II) complexes with various structural properties. Among the modifications are some simple chemical modifications, the combination of complexes with organic small molecules, the introduction of chiral groups and the incorporation of some ligands with anticancer and antibacterial activities [36]. Antibacterial ruthenium(II) metal complexes provide the following characteristics [37,38,39,40,41,42,43]: (1) Carrier. The metal center acts as a carrier for active ligands (usually drugs) that enhance drug activity by temporarily coordinating with the metal portion. (2) Catalyst. Metal complexes act as catalysts, that is, substituting inert complexes can catalyze redox cycles and oxidize glutathione (GSH) to glutathione disulfide (GSSG), resulting in a significant increase in reactive oxygen species (ROS) and high cytotoxicity to bacteria. (3) Photosensitizer. Metal complexes are photoactive and can be used as photosensitizers, and polypyridine ruthenium(II) complexes can retain the ability to produce singlet oxygen due to their intense visible light absorption and abundant excited state properties [44,45]. The long-term trilinear excited states of Ru(II) complexes exhibit particularly robust redox properties and can facilitate intermolecular electron transfer or energy transfer [46,47,48]. Therefore, Ruthenium complexes can serve as potential candidates for photodynamic therapy (PDT) against diseases caused by microbial pathogens. Photodynamic therapy (PDT) is mainly performed by two mechanisms, SN-I or SN-II [11]. The SN-I mechanism is where the activated PS contacts directly with biomolecules and then reacts to yield free radicals which can eventually kill bacteria or react with oxygen molecules to produce superoxide ions (•O^2-^) or hydroxyl radicals (•OH). The SN-II mechanism involves the interaction of the excited PS with the ground state oxygen molecule (^3^O_2_) to release the extremely toxic singlet oxygen (^1^O_2_). In this study, the SN-II monoclinic oxygen mechanism is considered to be predominant.

**Figure 1:**
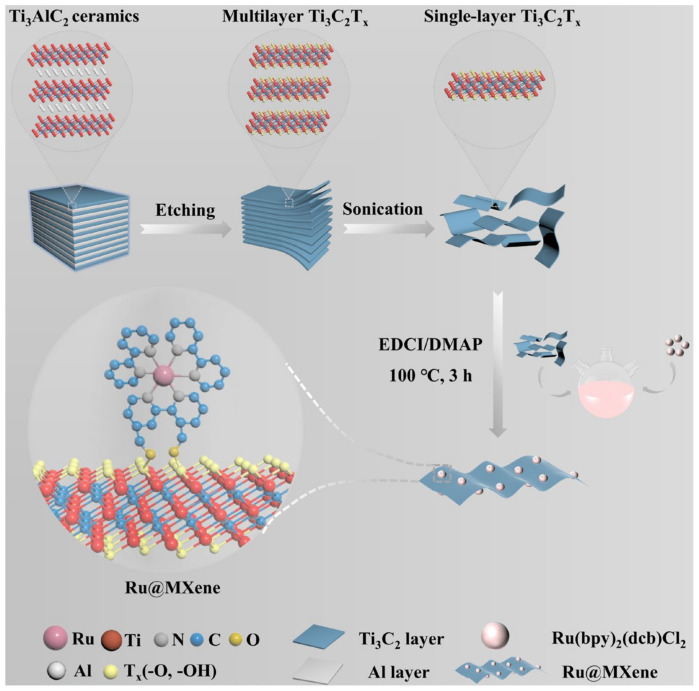
Schematic illustration of the preparation of Ru@MXene nanocomposites.

**Figure 2:**
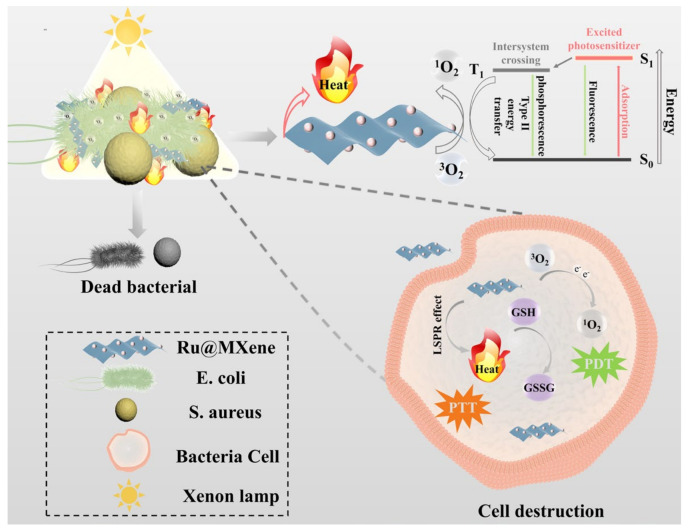
Schematic diagram of Ru@MXene as a photoconverting agent to produce heat and ^1^O_2_ for achieving PTT/PDT antibacterial activity.

Inspired by above-mentioned considerations, and in order to solve the issue of bacterial resistance to antibiotics and the poor therapeutic effect of photosensitizers or nanomaterials alone, a synergistic photoinduced photothermal/photodynamic therapy (PTT/PDT) antimicrobial strategy was investigated. The ruthenium complex and MXene are self-assembled to form a covalent bond through a surface functional group reaction, such that the ruthenium complex is loaded onto the surface of MXene to produce the Ru@MXene nanocomposite system (Figure 1). Therefore, a synergistic antibacterial system with three-in-one antibacterial mechanism, including ultra-thin MXene nanosheets physically cutting cell membranes, PTT and PDT, can be achieved, which provides a new idea for alternative antibiotic therapy (Figure 2).

## 2. Experimental Methods

### 2.1. Materials

RuCl_3_-3H_2_O, Dimethyl sulfoxide (DMSO, 99.9%, AR), 5,5′-Dithiobis (2-nitrobenzoic acid) (DTNB, 98%, AR), Glutathione (Reduced) (GSH, 98%, AR), 2,7-Dichlorodihydrofluorescein diacetate (DCFH-DA, 99%) and 4-Dimethylaminopyridine (DMAP, 99%) were supplied by Aladdin company (Shanghai, China). 2,2′-bpyrine (bpy, 99%, AR), 1,3-diphenylbenzofuran (DPBF, 97%, AR), 2.5% Glutaraldehyde Solution, N-(3-dimethylaminopropyl)-N′-ethylcarbodiimide hydrochloride (EDCI, 98.5%), N,N-Dimethylformamide (DMF, 99.5%, AR), Lithium chloride (LiCl, 99%) and Lithium fluoride (LiF, 99%, AR) were supplied by Macklin company (Shanghai, China). 2,2-bipyridine-4,4 dicarboxylic acid (98%) was supplied by Energy Chemical company (Shanghai, China). Ti_3_AlC_2_ (MAX, 200 mesh, 98%) was supplied by Laizhou Kai Kai Ceramic Materials Co., Ltd. (Shandong, China). Hydrogen peroxide (H_2_O_2_, 30 wt%) and Hydrogen chloride (HCl, 37 wt%) was from Guangzhou Chemical Reagent Factory (Guangzhou, China).

### 2.2. Characterization

Field emission scanning electron microscopy (FE-SEM, HT7700, Hitachi, Tokyo, Japan) was applied to measure the micromorphology of samples and the elements content of loading with an acceleration voltage of 15 kV at a magnification of 20,000×. Scanning electron microscopy (SEM, Phenom World ProX, Eindhoven, The Netherlands) was applied to observe the macroscopic morphology of multi-layer MXene with accelerating voltages of 15 kV at a magnification of 20,000×. The SEM samples were dispersed in ethanol, dropped on silicon wafers, dried at room temperature and pasted on conductive adhesive and the surface morphology of the samples was observed after spraying gold on the sample surface. The transmission electron microscope (TEM, HT7700, Tokyo, Japan) was used to observe the microstructures of samples and bacteria with accelerating voltages of 120 kV at a magnification of 8000×. The X-ray diffractometer (XRD, MiniFlex 600, Rigaku, Tokyo, Japan) applied to measure the crystallization of the sample was equipped with a Cu-Kα tube and a Ni filter (λ = 0.1542 nm) and operated at 40 kV, a current of 30 mA, a sweep speed of 5°/min and a test scan angle of 5° to 80°. The stripping state and thickness of MXene were evaluated by atomic force microscopy (AFM, Bruker Dimension FastScan, Karlsruhe, Germany). The X-ray photoelectron spectroscope (XPS, Escalab 250Xi, Thermo Fisher, Waltham, MA, USA) was used to test the elements’ compositions. Fourier transform infrared (FTIR) spectra measurements were performed with a resolution of 4 cm^−1^ and a range from 400 to 4000 cm^−1^ with 16 scans (Thermo Fisher Nicolet 6700, Waltham, MA USA). Nano particle size and Potential analyzer (BI-200SM Brookhaven, Brookhaven, Holtsville, NY, USA,) was applied to measure the potential of the sample. The ultraviolet–visible (UV–vis) absorption spectra was tested by UV–vis spectrophotometry (Lambda 950, PerkinElmer, Waltham, MA, USA) to determine the absorption of light by the sample. Photoluminescence (PL) spectra were recorded on a HORIBA spectrometer (Fluorolog-3, Edison, NJ, USA). Ultra-high-performance liquid chromatography (UPLC) ESI mass spectrometry was performed on a Shimadzu LCMS-2020 system (Shimadzu, Kyoto, Japan). The NMR structures were analyzed by a Bruker AVANCE III 400 MHz Superconducting Fourier spectrometer (NMR, Bruker, Fällanden, Switzerland). The bacteria were observed with an Airscan laser confocal microscope (Carl Zeiss, Jena, Germany) and an inverted fluorescence microscope (Olympus, Tokyo, Japan). The photothermal performance tests were conducted under the simulated sunlight provided by the xenon lamp (CEAULIGHT, CEL-HXUV300, Beijing, China). The infrared thermal imager (MAGNITY MAG12, Shanghai, China) was applied to record the change of sample temperature with time during the measurement. The photodynamic property measures were conducted under the simulated sunlight provided by the 532 nm semiconductor lasers (MW-SL-532, Changchun Laser Optoelectronics, Changchun, China).

### 2.3. Ti_3_C_2_T_x_ MXene Preparation

The MXene nanosheets were prepared according to the previously reported procedure, which was developed to produce high yields of the MXene nanosheets [49]. Briefly, the HCl/LiF etchant was processed by adding 3.2 g LiF to 40 mL 12 M HCl. Next, 2 g Ti_3_AlC_2_ ceramic powders (200 mesh) were slowly added to the above mixture and stirred continuously at 40 °C for 48 h. Afterward, the resultant Ti_3_C_2_T_x_ suspension was rinsed with deionized water and centrifuged for 5 min until the pH of the supernatant was approximately 7. The obtained Ti_3_C_2_T_x_ sludge was dispersed in deionized water and treated by sonication and shaking in an ice bath for 3 h. The mixture was centrifuged for 1 h to obtain a supernatant with a monolayer of the Ti_3_C_2_T_x_ nanosheet and dried by vacuum freezing.

### 2.4. Synthesis of cis-[Ru(bpy)_2_Cl_2_]·2H_2_O

A suspension of RuCl_3_-3H_2_O, 2,2′-bpyrine (bpy) and LiCl in DMF was heated and refluxed under argon atmosphere for 12 h. The mixture was cooled to room temperature and acetone was added. Subsequently, the mixture was stored overnight at −20 °C to obtain crude dark green crystals. The precipitate was filtered and washed with ice water and acetone until the filtrate was almost colorless. Eventually, the crystals were dried in vacuum dryers to give a good yield of pure dark green cis-[Ru(bpy)_2_Cl_2_]·2H_2_O crystals.

### 2.5. Synthesis of Ru(bpy)_2_(dcb)Cl_2_

Cis-[Ru(bpy)_2_Cl_2_]·2H_2_O and 2,2-bipyridine-4,4 dicarboxylic acid were added in 50 mL of ethanol and the pH adjusted to be alkaline with 1 M NaOH. The mixture was dissolved and refluxed at 80 °C for 6 h under the argon atmosphere. When the reaction was completed, the pH was adjusted to be acidic, and the solvent was removed. Subsequently, the precipitate was removed by successive centrifugation with water and ethanol. The clarified filtrate was filtered and dried in a vacuum desiccator to obtain orange-red crystals Ru(bpy)_2_(dcb)Cl_2_. ESI-MS (CH_3_OH): *m*/*z*: 329.04 (Figure S4). 1H NMR (400 MHz, DMSO): δ 8.85 (d, J = 8.2 Hz, 4H), 8.79 (s, 2H), 8.15 (dd, J = 13.0, 6.6 Hz, 4H), 7.74 (d, J = 5.4 Hz, 6H), 7.66 (d, J = 5.7 Hz, 2H), 7.53 (t, J = 6.7 Hz, 4H) (Figure S3).

### 2.6. Fabrication of Ru@MXene Nanocomposites

The MXene nanosheets were further modified by esterification between the carboxyl group of Ru(bpy)_2_(dcb)Cl_2_ and the hydroxyl group of MXene nanosheets [50]. A uniformly dispersed MXene suspension was obtained by ultrasonication in an ice bath for 30 min and Ru(bpy)_2_(dcb)Cl_2_ was added. A water-soluble composite catalyst composed of EDCI and DMAP (mass ratio 1:1) was added to facilitate the reaction. The reaction was carried out at 100 °C for 3 h. The mixture was repeatedly washed with deionized water and centrifuged several times. Finally, the product was freeze-dried to obtain Ru@MXene.

### 2.7. Load of Ru on Ru@MXene

The fluorescence spectrum of the Ru solutions at concentrations of 0.0625, 0.125, 0.25, 0.5, 1, and 2 μM was determined using a fluorescence spectrometer, and these data were used to construct standard curves of Ru solutions [51,52]. Briefly, 8 μL of a 5 mg/mL Ru@MXene dispersion was added to 10 wt% NaOH solution at a final concentration of 10 μg/mL. The mixture was incubated in a shaking incubator for 24 h to break ester bonds. The mixture was filtered, and the supernatant liquid was used to check the fluorescence spectrum of Ru(bpy)(dcb)Cl_2_. This was employed to determine the content of Ru in solution, calculated using the standard curve (Figure S5).

### 2.8. Detection of ROS

The UV spectrum of 1,3-diphenylbenzofuran (DPBF) used as a monoclinic oxygen (^1^O_2_) trapping agent has a maximum absorption peak at 417 nm, while its oxidation by monoclinic oxygen leads to a lower absorption peak here. The entire test was carried out in a quartz cuvette in a dark environment. Briefly, 24 μL of DPBF (10 mM, dissolved in DMSO) was added to MXene/DMSO, Ru/DMSO, Ru@MXene/DMSO and DMSO. The solutions were then irradiated using a 532 nm LED lamp (laser power: 20 mw). The absorption spectra of the mixtures after 532 nm laser irradiation were measured using a UV–Vis spectrometer. the DPBF/DMSO group was used as a control experiment.

### 2.9. Photothermal Experiments

Xenon lamps were used to irradiate different concentrations of Ru@MXene dispersions (0, 20, 50, 100 μg/mL) and different samples (Water, MXene, Ru, Ru@MXene) for 30 min, while the real-time temperature of the samples was recorded and photothermal images were obtained at intervals by infrared thermal imagers. The thermal stability of the sample was tested by cyclic heating and cooling tests, where the light source was removed after a heating cycle had been completed until it cooled to room temperature, then the light source was lit again; this process was repeated for 5 cycles. Meanwhile, an infrared thermal imager recorded the real-time temperature of the sample. A xenon lamp equipped with an AM 1.5 G filter (150 mW/cm^2^) was used as the light source.

### 2.10. Antibacterial Activity Assessments

*Escherichia coli* (*E. coli*) and *Staphylococcus aureus* (*S. aureus*) were used as bacterial models. The antibacterial activities of the samples were evaluated by the plate-counting approach, Live/Dead staining and the TEM observation of bacteria. The bacteria were cultured in LB medium at 37 °C for 12 h. Afterwards, the cell solution was centrifuged at 5000 rpm for 5 min, and the precipitate obtained was washed with sterile water three times. Finally, the cell precipitate was resuspended in sterile water and diluted to an approximate cell concentration of 10^7^ CFU/mL. The bacterial solution was mixed with the materials (materials’ final concentration: 110 μg/mL for Ru@MXene, 15 μm for Ru, and 100 μg/mL for MXene. The concentrations of Ru and MXene are consistent with the ratio in Ru@MXene) and incubated at 37 °C for 1 h. The mixture was separated into a light group and a dark group. The light group was irradiated with a xenon lamp for 30 min while the dark group was untreated. A xenon lamp equipped with an AM 1.5 G filter (150 mW/cm^2^) was used as the light source. Next, 100 μL of the mixture was dispensed onto the solid medium and spread well. Finally, plates were incubated at 37 °C for 24 h. The experiments were carried out using the bacterial solution without any materials as a blank control.

The TEM can more directly and clearly observe the changes of bacterial morphology. First, the bacterial suspension was mixed with the material and incubated for 1 h at 37 °C with shaking. The cells were washed three times with water, collected by centrifugation and then fixed in 2.5% paraformaldehyde fixative for 5 h. The supernatant was discarded by centrifugation at low speed. The cells were dehydrated in a gradient of 20%, 30%, 50%, 70%, 90% and 100% ethanol for 15 min. Finally, the morphology of the treated bacteria was observed by transmission electron microscopy.

Bacterial live/dead staining was also employed to test the antimicrobial activity of the samples. Following light exposure, propidium iodide (PI) and acridine orange (AO) were utilized to stain the bacteria (AO marked live bacteria as green and PI marked dead bacteria as red). The staining procedure took roughly 20–30 min. The dye was subsequently removed and the cells washed twice with PBS. The final observation was performed by Laser Confocal Microscopy.

Reactive oxygen species (ROS) detection was undertaken using a reactive oxygen species detection kit. Samples in the light and dark groups after treatment were incubated with 10 μM 2,7-dichlorodihydrofluorescein diacetate (DCFH-DA) for 20–30 min and washed with PBS. Bacterial cells were inspected using a fluorescent inverted microscope.

### 2.11. Ellman’s Assay

The materials were mixed with GSH solution (50 μL, 0.8 mM) and then incubated in the dark for 10 min to reach an equilibrium between adsorption and desorption. The mixture was then exposed to a xenon lamp (150 mW/cm^2^, 30 min) or treated in the dark. Subsequent to treatment, phosphate buffer solution (800 μL, pH = 8) and DTNB solution (20 μL, 10 mM) was added to the mixture. The mixture was filtered to remove the material. Finally, the supernatant absorbance was measured with a UV–Vis spectrophotometer at 412 nm. H_2_O_2_ and GSH were established as positive and negative controls, respectively.

## 3. Results and Discussions

### 3.1. Morphological Observations

**Figure 3:**
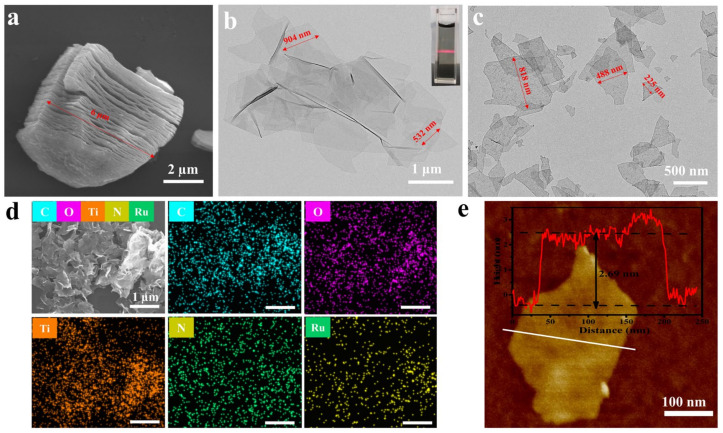
(**a**) SEM micrographs of multilayered Ti_3_C_2_T_x_. (**b**) TEM images of single-layer Ti_3_C_2_T_x_ nanosheets. Inset shows a digital photo of Ti_3_C_2_T_x_ nanosheets dispersed in water exhibiting the Tyndall effect when a red laser beam was used. (**c**) TEM images of Ru@MXene. (**d**) SEM images of Ru@MXene and corresponding elemental mapping. (**e**) AFM views of single-layer Ti_3_C_2_T_x_ nanosheets.

To address the bacterial resistance barrier, a novel Ru@MXene 2D nanoplatform with photothermal/photodynamic synergistic antimicrobial properties has been designed and developed as shown in Figure 1 and Figure 2. Initially, Ti_3_AlC_2_ ceramics were etched to remove the aluminum layer, utilizing a HCl/LiF solution to obtain multilayer Ti_3_C_2_T_x_, and ultra-thin Ti_3_C_2_T_x_ nanosheets were obtained by means of ultrasonication. Subsequently, ruthenium complexes with photodynamic properties were grafted onto the Ti_3_C_2_T_x_ surface, and eventually the nanocomposite Ru@MXene with both photothermal and photodynamic properties was obtained (Figure 1). In order to observe the surface morphology and spatial structure of the prepared nanomaterials, SEM, TEM, Mapping and AFM were selected for characterization. The SEM image in the Figure 3a shows that the MAX phase Ti_3_AlC_2_ has been etched with HF (LiF + HCl) and has changed from a stacked structure to a unique accordion-like shape with a clearer and more defined layer structure. The TEM micrograph clearly shows that both Ti_3_C_2_T_x_ (Figure 3b) and Ru@MXene (Figure 3c) have translucent images with clear edges and unique shapes. The AFM pattern of the MXene nanosheets is illustrated in Figure 3e. It is evident that the area marked by the white line has a uniform height distribution between approximately 2.6–3.1 nm, which corresponds to a Ti_3_C_2_T_x_ monolayer of roughly 2.7 nm [53]. These imply that MXene has an ultra-thin structure. Subsequently, the elemental mapping (Figure 3d) revealed a dispersed distribution of N and Ru elements in Ru@MXene, proving the presence of Ru(bpy)_2_(dcb)^2+^ and the successful immobilization of Ru(bpy)_2_(dcb)^2+^ on the surface of the MXene nanosheets. Moreover, the well-dispersed Ti and C elements are evidence that MXene is a carrier for Ru(bpy)_2_(dcb)^2+^.

### 3.2. Chemical Structure Analysis

**Figure 4:**
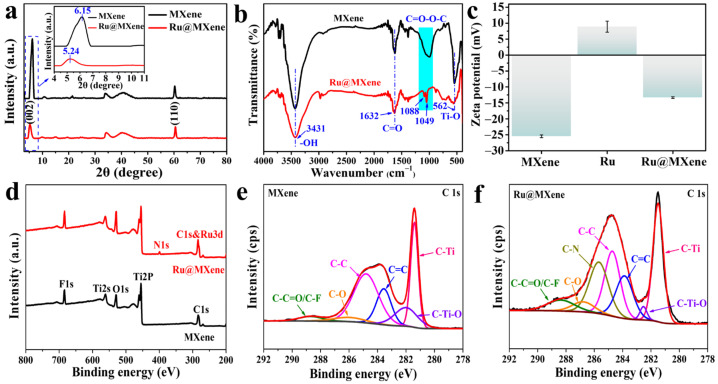
(**a**) XRD and (**b**) FTIR spectra of MXene and as-prepared Ru@MXene. (**c**) Zeta potential of MXene, Ru and Ru@MXene. The error bars indicate mean ± SD (n = 3). X-ray photoelectron spectra analyses of MXene and as-prepared Ru@MXene: survey scan spectrumof (**d**) both MXene and Ru@MXene; (**e**) MXene, C 1s; and (**f**) Ru@MXene, C 1s.

The chemical structures of MXene, and Ru@MXene were confirmed by XRD, FT-IR, ζ-potential and XPS. X-ray diffraction (XRD) was used to analyze the crystal structures of Ti_3_AlC_2_, Ti_3_C_2_T_x_ and Ru@MXene. As shown in Figure S1, the (104) peak of Ti_3_AlC_2_ disappears in the XRD pattern of Ti_3_C_2_T_x_, indicating the successful removal of the Al layer after etching. The (002) peak of the multilayered Ti_3_C_2_T_x_ shifts to a lower 2θ degree due to the increased layer spacing compared to the stacked Ti_3_AlC_2_ [50]. As shown in Figure 4a, it is noteworthy that the (002) peak of Ru@MXene is further shifted to a lower angle of 0.91° compared to the multilayer Ti_3_C_2_T_x_, corresponding to a remarkable increase in the layer-to-layer spacing of Ti_3_C_2_T_x_ [54] ascribed to the super-thin nanosheet fabrication.

As can be seen from the IR spectra in Figure 4b, both MXene and Ru@MXene show typical characteristic bands at 3431, 1632 and 562 cm^−1^, which correspond to the stretching vibrations of -OH, C=O and Ti-O, respectively. After modification with ruthenium complexes, absorption bands associated with ester bonds appear at 1088 and 1049 cm^−1^ [54,55], indicating successful esterification between Ru-COOH and the MXene nanosheets. The mapping results are consistent with the FT-IR results, that is, both show that Ru(bpy)_2_(dcb)^2+^ has been modified on the surface of MXene. More specifically, Ru(bpy)_2_(dcb)^2+^ is adsorbed onto MXene via ester bonding and electrostatic interaction. In addition, the ζ-potential was used to demonstrate the electrostatic interaction between MXene and Ru(bpy)_2_(dcb)^2+^. The zeta potential data in Figure 4c authenticate that MXene exhibits a robust negative charge of −25.5 mV, allowing MXene to be extremely stabilized in aqueous solution. As a result, during the synthetic stage of Ru@MXene, the surface of MXene is electronegative and may readily absorb Ru(bpy)_2_(dcb)^2+^ cations by electrostatic effects. The ζ-potential was turned from −25.5 to −13.3 mV via grafting Ru(bpy)_2_(dcb)^2+^ onto MXene, since the electronegative MXene was neutralized by Ru(bpy)_2_(dcb)^2+^. In contrast, Ru(bpy)_2_(dcb)^2+^ exhibited an opposite charge of 9.0 mV. Therefore, Ru@MXene tended to be electrically neutral after chemical bonding of MXene and Ru(bpy)_2_(dcb)^2+^. These are obvious evidence for the successful preparation of Ru@MXene composites. The formation of the ester linkage and chemical structure variation of Ru-MXenes is also further supported by X-ray photoelectron spectroscopy (XPS). The survey scan spectrum of Ru@MXene in Figure 4d exhibits a new N1s peak and increased O1s peak intensity compared with pure MXene, which is ascribed to the N and O elements presenting in Ru(bpy)_2_(dcb)^2+^. The chemical bond variations were further revealed by the different chemical coupling state of C atoms. As shown in Figure 4e, the C 1 s core-level pattern could be curve-fitted with six peak components, including 281.4 (C-Ti), 282.1 (C=C), 283.6 eV (C-Ti-O), 284.8 (C-C), 286.2 (C-O), and 288.7 (C-C=O/C-F) [50,56]. After modification by Ru(bpy)_2_(dcb)^2+^, a new-emerging peak at approximately 285.6 eV assigned to a C-N bond and the augmented C=O/O-C=O peak intensities in C 1 s spectra of Ru@MXene (Figure 4f) illustrate the successful esterification reaction between the hydroxy groups of MXene nanosheets and carboxyl groups of serine molecules. The UV–Vis absorption spectra of the Ru(II) complexes in aqueous solution at room temperature are shown Figure S2. The strong and sharp absorption bands in the UV region of the prepared Ru(II) complexes are attributed to electron transfer leaps within the C^N ligand and the N^N ligand. The strong absorption of Ru(bpy)_2_(dcb)^2+^ at 400–500 nm indicates its efficient photon absorption and utilization. In spite of the fact that TGA analysis is a valid approach to determine drug loading, we have established our drug loading via the fluorescence intensity measuring methods and a standard curve. According to the calculation, the concentration of Ru in MXene is 1.39 μmol/L (Figure S5).

### 3.3. Photodynamic Properties

**Figure 5:**
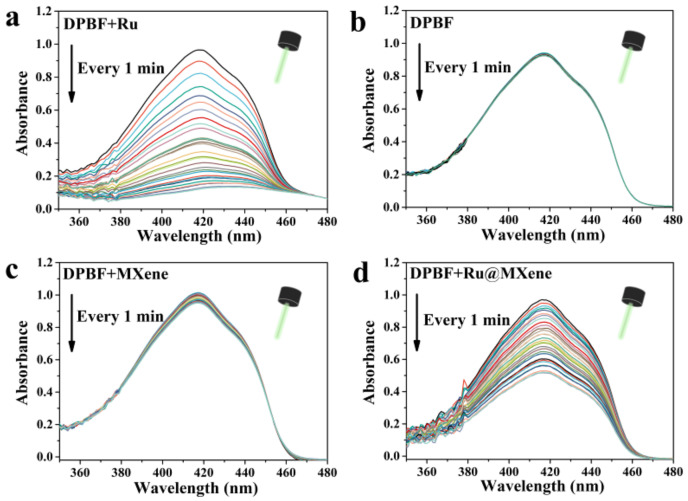
Decrease in absorbance spectra of DPBF for the detection of ^1^O_2_ in the presence of Ru (**a**); DPBF (**b**); MXene (**c**); and Ru@MXene (**d**) with 532 nm LED lamp illumination (20 mw).

To evaluate the photodynamic properties of the materials, DPBF was employed as the ROS trapping agent to determine their ability to generate singly linear oxygen (^1^O_2_) under 532 nm laser irradiation (20 mw). If DPBF is oxidized by ROS, its UV absorption peak at 417 nm will diminish. Figure 5 shows the changes in UV absorption under 532 nm laser irradiation for pure DPBF, Ru/DPBF, MXene/DPBF and Ru@MXene/DPBF. Figure 5a of the control group (pure DPBF) showed almost no change in its absorption peak under 532 nm LED lamp illumination for 30 min, indicating that pure DPBF does not produce singlet oxygen. The absorbance at 417 nm of MXene/DPBF group only decreased by 8.14%, implying that the photodynamic performance of MXene is poor, as shown in Figure 5c. In contrast, the maximum absorption peak of DPBF decreased by 87.73% after light exposure for the Ru/DPBF group in Figure 5b, revealing that Ru has excellent photodynamic properties and is able to generate more singlet oxygen at the same intensity and illumination time. Significantly, the absorbance of the Ru@MXene (Figure 5d) dropped by almost half. Ru@MXene generated reactive oxygen species at a rate slower than that of free Ru, though both yielded their own optimum amounts of reactive oxygen species, illustrating that incorporating Ru onto MXene nanosheets gives the Ru@MXene composite systems that preserve the capacity for the creation of reactive oxygen species by Ru alone. The modified Ru@MXene has a fall in the efficiency of ^1^O_2_ compared with Ru, which may be due to the shielding effect of MXene flakes. These phenomena can be interpreted through an in-depth insight into the photosensitization process of singlet production. There are three stages of the photosensitization procedure for PDT as shown in Figure 2. As soon as Ru has an electronic transition energy equivalent to that of the incident photon, absorption occurs. Electrons are shifted from the ground singlet state (S0) to an excited singlet state (S1) with higher energy [57]. Excited electrons from S1 transit to the excited triplet state (T1) via altering the orientation of the electron spin in the non-radiative inter-system crossing (ISC) pathway (II) [58]. The singlet oxygen (^1^O_2_) originates through an energy transfer route involving collisions from T1 to molecular oxygen (III) with a Type II mechanism [59].

### 3.4. Photothermal Properties

**Figure 6:**
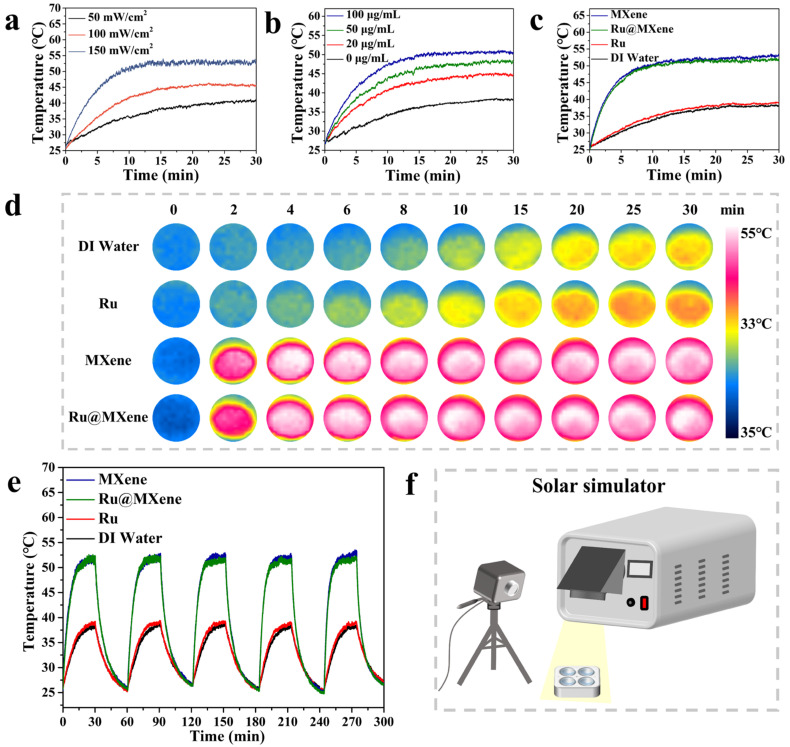
Photothermal property assessments: photothermal heating curve of Ru@MXene (**a**) with gradient concentrations; and (**b**) with different power density. (**c**) Photothermal heating curve of various samples with a power density of 150 mW/cm^2^. (**d**) Real-time infrared thermal images of different samples. (**e**) Photothermal periodic curve of Ru@MXene. (**f**) Schematic diagram of simulated sunlight irradiation samples.

The photothermal properties of the samples were assessed using a xenon lamp light source that simulates sunlight, and a schematic model of the irradiated samples is shown in Figure 6f [60]. To assess the effect of optical power density on the photothermal properties, the Ru@MXene dispersion (110 μg/mL) was exposed to a gradient of power (50, 100, 150 mW/cm^2^). As shown in Figure 6a, it can be clearly seen that the 50 mW/cm^2^ group showed an increase in minimum temperature from 26.9 °C to 40.3 °C. As the optical power density increased to 100 mW/cm^2^, the maximum temperature increased to 45.4 °C. Meanwhile, the 150 mW/cm^2^ group reached a maximum temperature of 53.2 °C. Therefore, high-power intensity will raise the final temperature of the materials under xenon lamp illumination. After exploring the link between power intensity and photothermal properties, Ru@MXene solutions (0, 20, 50 and 100 μg/mL) of gradient concentrations were individually exposed to the xenon lamp (150 mW/cm^2^) to investigate the relationship between photothermal properties and sample concentration, as shown in Figure 6b. Final temperatures were improved from 26.9 to 38.0, 45.3, 48.0 and 50.4 °C for the 0, 20, 50 and 100 μg/mL concentration groups, respectively. Benefiting mainly from the broad solar spectral absorption and the localized surface plasmon resonance (LSPR) effect of MXene, the MXene nanomaterial has excellent photothermal conversion properties and is able to collect and convert solar energy efficiently [61]. Furthermore, the optical power density (L) is almost linearly related to the saturation temperature (T), as can be seen from the S-plot (Figure S6). To further demonstrate the photothermal effect of Ru@MXene, three samples of Ru (15 μM), MXene (100 μg/mL) and Ru@MXene (110 μg/mL) were employed for comparative photothermal testing and the results are shown in Figure 6c. These concentrations were calculated corresponding to the Ru loadings. The heating process can be visualized from the photothermal image in Figure 6d. It is evident that both MXene and Ru@MXene exhibit excellent photothermal properties compared with the Ru(II) complex. This is due to the laminar structure of MXene resulting in a continuous thermal conductivity interface, which enhances the thermal performance of Ru@MXene. Furthermore, the temperature change profile (Figure 6e) for five cycles under constant illumination of 150 mW/cm^2^ is essentially the same, also confirming the cyclically stable heating performance of Ru@MXene as a photothermal therapeutic material. Thus, all the above results and mechanisms confirm the potential of Ru@MXene as a photothermal antimicrobial agent.

### 3.5. Photoinactivation of E. coli and S. aureus

As stated in the introduction, we assumed that high heat and ROS generated by light could inhibit the growth of bacteria. Therefore, the antibacterial performance of Ru, MXene, Ru@MXene materials was evaluated by the flat-counting method in the dark and under illumination, respectively. *Escherichia coli* (Gram-negative) and *Staphylococcus aureus* (Gram-positive) bacteria were made available as the model bacteria, and xenon lamps provided the light source. The minimum bactericidal concentration (MBC) of the samples was studied according to the protocol described elsewhere with appropriate adjustments (Figure S7) [27,62]. Bacterial colony photos (Table S1) recorded MBC values of 200 μg/mL for *E. coli* and 100 μg/mL for *S. aureus*, while the bacteria survival rate of *E. coli* and *S. aureus* treated with Ru, MXene and Ru@MXene under dark and light conditions gradually declined as depicted in Figure S8.

**Figure 7:**
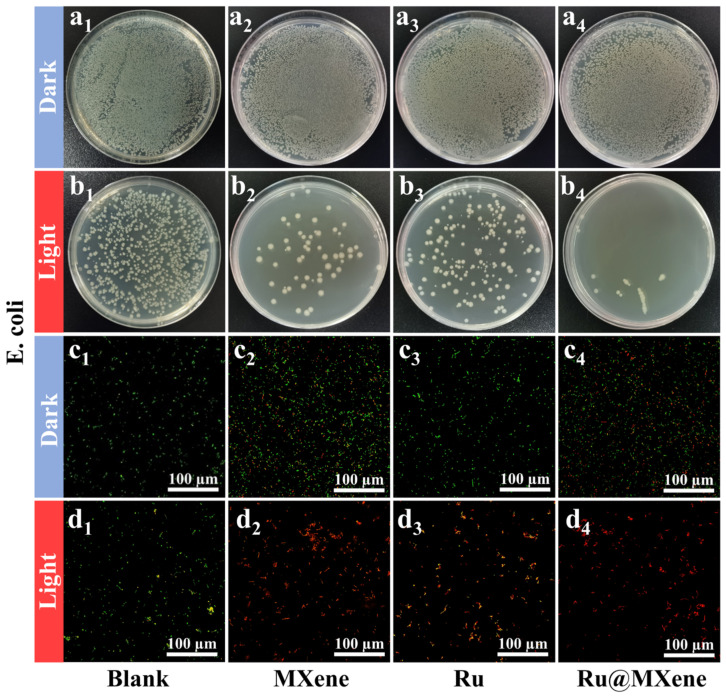
Antibacterial activities: bacterial suspensions without any material were used as control (**a**_1_–**d**_1_). Spread plate results of *E. coli* treated with MXene (**a**_2_,**b**_2_), Ru (**a**_3_,**b**_3_) and Ru@MXene (**a**_4_,**b**_4_) after exposure in the dark or illumination with xenon lamp (150 mW/cm^2^, 30 min). Corresponding confocal images of live/dead fluorescence of *E. coli* on MXene (**c**_2_,**d**_2_), Ru (**c**_3_,**d**_3_) and Ru@MXene (**c**_4_,**d**_4_) with or without xenon lamp (150 mW/cm^2^, 30 min). (Sample concentration: 110 μg/mL for Ru@MXene, 15 μm for Ru, and 100 μg/mL for MXene. The concentrations of Ru and MXene are consistent with the ratio in Ru@MXene).

**Figure 8:**
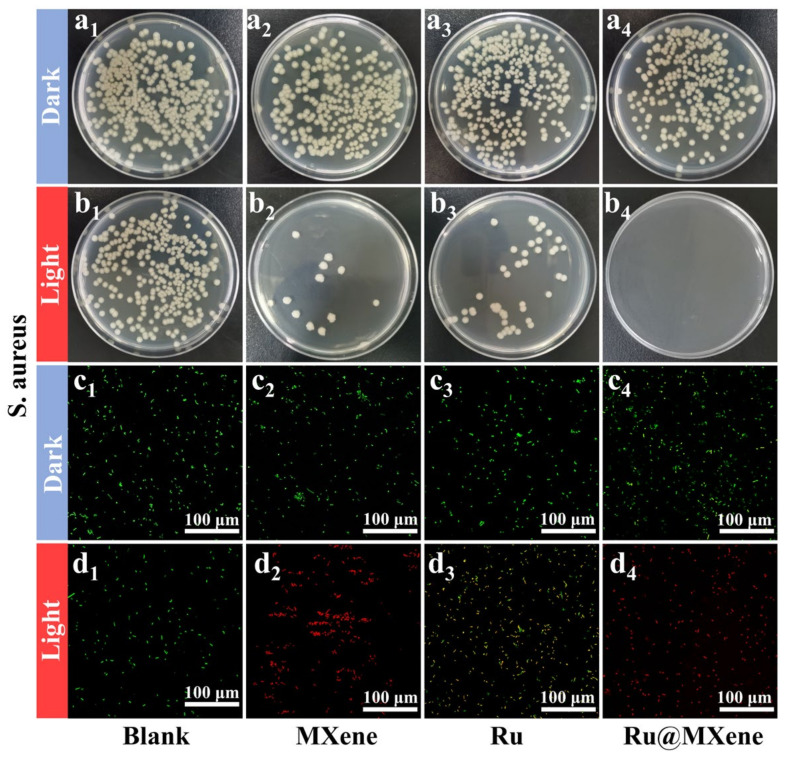
Antibacterial activities: bacterial suspensions without any material were used as control (**a**_1_–**d**_1_). Spread plate results of *S. aureus* treated with MXene (**a**_2_,**b**_2_), Ru (**a**_3_,**b**_3_) and Ru@MXene (**a**_4_,**b**_4_) after exposure in the dark or illumination with xenon lamp (150 mW/cm^2^, 30 min). Corresponding confocal images of live/dead fluorescence of *S. aureus* on MXene (**c**_2_,**d**_2_), Ru (**c**_3_,**d**_3_) and Ru@MXene (**c**_4_,**d**_4_) with or without xenon lamp (150 mW/cm^2^, 30 min).

It was revealed that Ru@MXene had exhibited excellent light-activated antibacterial capabilities against both Gram-positive and negative bacteria in the presence of light. The photoactive antibacterial effects of materials (Ru, MXene and Ru@MXene) via the flat-counting method under dark and light conditions were investigated as depicted in Figure 7 (*E. coli*: dark group (a1–a4), light group (b1–b4)) and Figure 8 (*S. aureus*: dark group (a1–a4), light group (b1–b4)).

MXene displayed favorable antibacterial activity against *E. coli* and *S. aureus* under light exposure, which is attributed to the optimal photothermal properties of MXene. The antibacterial test results were in accordance with the photothermal performances indicated in Figure 6. According to the temperature captured by the infrared thermography, the final surface temperature for both MXene and Ru@MXene dispersions reached 53.2 °C (Xenon lamp, 150 mw/cm^2^). However, normal cells and tissues are also damaged when the photothermal temperature of the bacteria is heated to over 50 °C [63]. The light group of Ru@MXene was noteworthy for its more pronounced bactericidal effect than the monotherapy group (Ru group or MXene group). The photodynamic properties of Ru@MXene in Figure 5d showed that the UV absorption of the DPBF in the Ru@MXene group dropped by almost 50% after 30 min illumination with the 532 nm laser, giving rise to a large amount of singlet oxygen, that is to say, the Ru@MXene composite system has excellent photodynamic antibacterial properties. This indicates that upon loading Ru on the surface of MXene sheet, the Ru@MXene composite system can generate a massive amount of singlet oxygen under illumination, and the antibacterial performance against *E. coli* and *S. aureus* is substantially improved. The Ru@MXene complex exhibits well-developed PTT/PDT synergistic antibacterial efficacy, owing to high temperature and ROS generation upon light exposure which are more likely accessible to bacteria. In other words, the attached bacteria are more vulnerable to be killed. Moreover, Ru@MXene proved to be more strongly antibacterial against *S. aureus* than *E. coli*, which is probably related to the structural variations in the cell walls of Gram-positive and Gram-negative bacteria [64]. A comparison of the antimicrobial efficiency among some PTT and PDT drugs are also listed. Table S2 includes conditions such as antimicrobial mechanism, nanomaterial concentration and antimicrobial activity [27,33,34,65,66,67,68,69,70]. It is observed that the antibacterial properties of MXene and its composites are outstanding under similar conditions.

To further explore the effect of light-activated antibacterial activity, live/dead fluorescence detection of *S. aureus* and *E. coli* was carried out using Acridine Orange (AO) and Propidium Iodide (PI). The confocal laser scanning microscope (CLSM) was made available to visualize the color of the stained bacteria. Figure 7 (*E. coli*: dark group (c1–c4), light group (d1–d4)) and Figure 8 (*S. aureus*: dark group (c1–c4), light group (d1–d4)) displayed the fluorescent staining results of the bacteria after being treated with the light and dark in the presence of materials (Ru, MXene, and Ru@MXene). A large proportion of bacteria in the blank group were stained with green fluorescence, implying that the majority of bacteria were still alive. *E. coli* and *S. aureus* that had been treated with Ru@MXene in the light group were stained red by PI (and *S. aureus* was more affected than *E. coli*). Consequently, treatment of bacteria with Ru@MXene in the light group led to almost all their death. The bacteria treated with Ru@MXene generated redder signals than MXene and Ru in the light group, indicating its superior antibacterial activity. This was in accordance with the results of previous tests. Overall, the results reveal that Ru@MXene possesses excellent photo-activated antimicrobial activity.

### 3.6. Investigation of the Mechanism of Synergistic Antibacterial Effect

**Figure 9:**
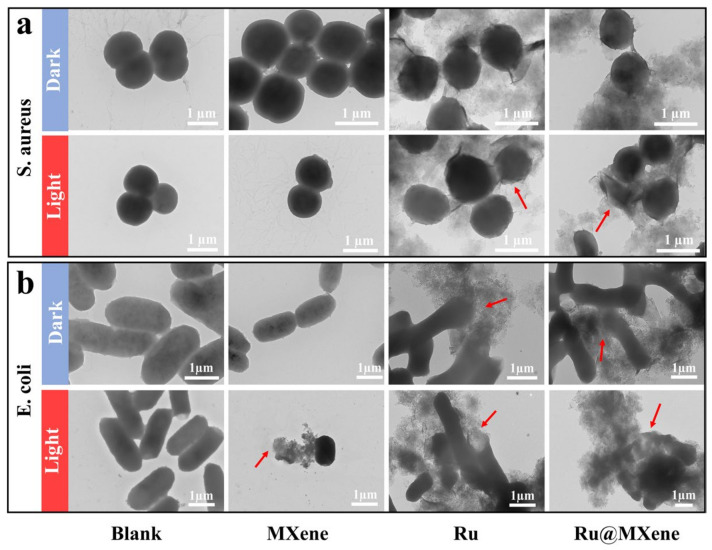
TEM images of bacterial structure and morphology of (**a**) *S. aureus* and (**b**) *E. coli* after being treated with MXene, Ru and Ru@MXene under dark or illumination. (xenon lamp, 150 mW/cm^2^, 30 min). Red arrows: disrupted bacterial walls.

TEM was utilized to visualize the variation in the morphology of both *E. coli* and *S. aureus* after treatment with materials. This experiment further confirms the ability of bacteria to survive after being treated with nanomaterials (but it also provides insight into the antibacterial mechanism of nanomaterials). As shown in Figure 9, *E. coli* and *S. aureus* showed varying degrees of deformation (marked by red arrows), while the *S. aureus* (spherical shape) wrapped in MXene or Ru@MXene nanosheets in dark conditions had a relatively smooth and well-integrated cell wall. After 30 min exposure to xenon light, the cell walls of *S. aureus* in contact with MXene and Ru@MXene were shrunken and deformed along with a variation in their size. In particular, the leakage of cell contents was observed in the majority of bacterial cells treated with Ru@MXene. This confirmed that bacteria were synergistically killed by the PTT/PDT effect within a short period of time, rendering the integrity of the cell wall and cytoplasmic membrane of both Gram-positive and Gram-negative bacteria heavily fragmented. Furthermore, the material has the capacity to break down cell membranes, allowing access to the inside of the cell, generating heat and ROS under light and eventually leading to self-destructive death.

**Figure 10:**
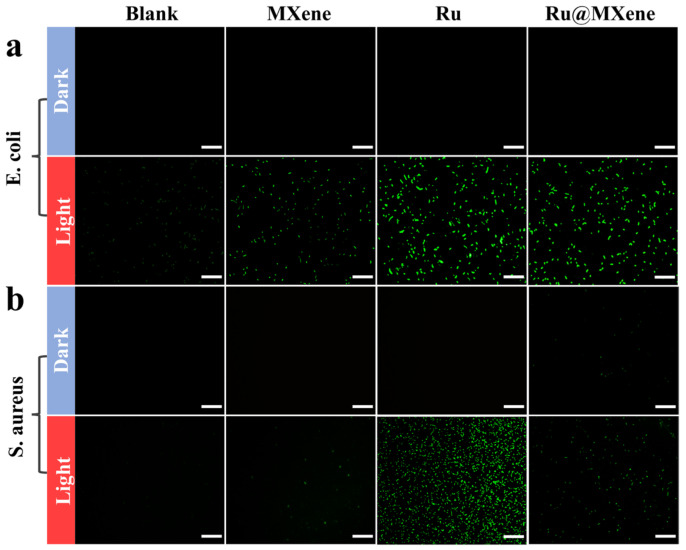
Antibacterial mechanism evaluations involving intracellular ROS level detection: Fluorescence intensity of intracellular ROS induced by different samples with or without xenon lamp illumination (150 mW/cm^2^, 30 min) in (**a**) *E. coli* and (**b**) *S. aureus*. (Scale bar: 50 μm).

To further explore what biological alterations occur upon entry of materials into cells causing bacterial death, the variation of ROS levels in bacteria and whether oxidative damage leads to bacterial death were investigated. Some antibiotics have been reported to kill bacteria through inducing ROS production and, in turn, inhibiting oxygenase activity. Therefore, the total ROS content was monitored by use of a reactive oxygen probe. It is shown in Figure 10 that when bacteria and materials were co-cultured for 1 h, the ROS levels in the light group treated with MXene still remained low, and those of *S. aureus* and *E. coli* treated with Ru and Ru@MXene were considerably raised. These results demonstrated that the antimicrobial activity of these materials was correlated with the ROS generation and consistent with previous in vitro testing of ROS levels in Figure 5. Ru@MXene in the light group induces high levels of ROS in bacterial cells due to its entry into the cell and disrupts the cell membrane of bacteria, causing oxidative damage and affecting the activity and function of macromolecules in bacteria, ultimately killing them and exhibiting high antimicrobial activity.

**Figure 11:**
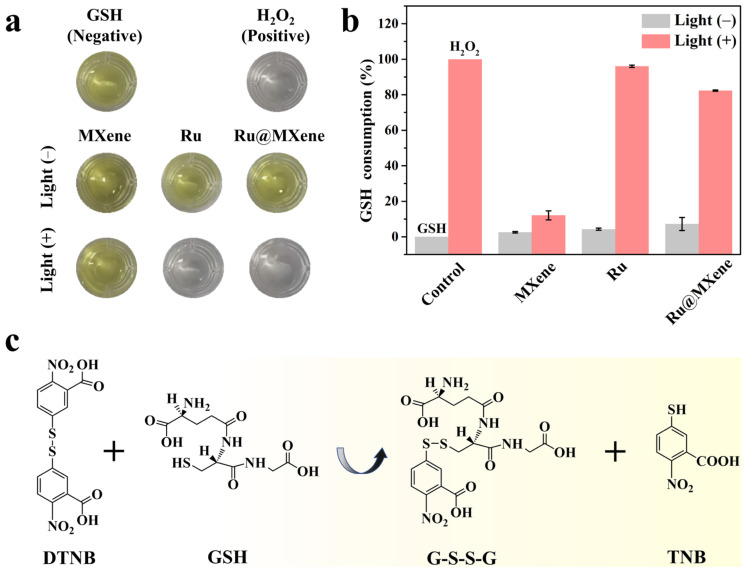
Antibacterial mechanism evaluations involving GSH consumption: (**a**) Photographs of the color change in GSH solution after incubating with various materials with or without xenon lamp illumination (150 mW/cm^2^, 30 min). (**b**) Corresponding GSH consumption rate (n = 3). (**c**) Chemical reaction between DTNB and GSH.

It is widely acknowledged that glutathione (GSH) is the predominant antioxidant sulfhydryl species in bacteria and serves an essential antioxidant role in the repair of oxidative protein damage [71]. It allows the material’s ROS production capacity to be tested indirectly by measuring the GSH consumption. The sulfhydryl group of GSH is able to react with 5,5′-dithiobis-(2-nitrobenzoic acid) (DTNB) to yield both yellow 2-nitro-5-mercaptobenzoic acid (TNB) and glutathione disulfide (GSSG), as shown in Figure 11c. TNB has a characteristic absorption peak at 412 nm and the GSH level can be quantified by the absorbance value variation. The color variations of the GSH solutions were photographed after being incubated with various materials in the presence or absence of light as shown in Figure 11a. The rate of GSH loss was calculated with absorption values as shown in Figure 11b. In the MXene group, almost no color alteration was observed with or without light, demonstrating the lack of oxidative activity of MXene alone, which is due to the fact that MXene only has PTT and PTT itself does not cause bacterial inactivation through oxidative stress. With respect to the Ru and Ru@MXene groups, the rate of GSH depletion in the dark was 2.57% and 7.22%, respectively. After illumination, the corresponding ratios increased further to 96.03% and 82.33%, as both had PDT, generating large amounts of ROS and thus oxidizing GSH. Accordingly, the outcomes of this assay prove that Ru@MXene has vigorous PDT effect and is capable of oxidizing GSH, the endogenous bacterial antioxidant, to boost oxidative stress.

## 4. Conclusions

In this work, we have successfully integrated Ru into MXene through covalent bonding to achieve light-modulated high PTT/PDT antibacterial performance against Gram-positive (*S. aureus*) and Gram-negative bacteria (*E. coli*). On the basis of various antimicrobial testing methods, the composite material Ru@MXene was observed to be extraordinarily effective in killing *E. coli* and *S. aureus*. The antibacterial mechanisms of Ru@MXene are attributed to four factors as follows: (1) Physical cutting of bacterial cell membranes by MXene nanosheets with their “knife-like edge”; (2) hyperthermia produced by photothermal effect of MXene; (3) ROS production (including ^1^O_2_) by Ru after exposure to xenon lamp illumination (150 mW/cm^2^, 30 min); and (4) the germicidal effect by adhering to bacteria with negative potential. Since MXene is covalently combined with Ru(bpy)_2_(dcb)^2+^ through hydroxyl groups, Ru@MXene obtains more positive charge due to charge neutralization, resulting in photoactivated sterilization. Heretofore, light-responsive antibacterial properties of MXene covalently bonded with ruthenium complexes coupling PTT and PDT properties has barely been reported. Overall, the Ru@MXene complex with light-controlled synergistic PTT/PDT antibacterial outcome provides a new forward-looking strategy to design light stimuli-responsive multifunction platforms to combat over-resistant bacterial. While the biocompatibility and toxicity of Ru@MXene were not deeply investigated in this study, based on previous studies of similar materials and analysis of the material’s chemical structure, it is expected that this nanocomposite material may have good biocompatibility and low toxicity to the environment. Future research is needed to further explore the biocompatibility and toxicity of the material to determine its feasibility for medical and environmental applications.

## Data Availability

Data are contained within the article.

## Supplementary Materials

The following supporting information can be downloaded at: https://www.mdpi.com/article/10.3390/nano13060958/s1. Figure S1: XRD spectra of Ti_3_AlC_2_ and Ti_3_C_2_T_x_. Figure S2: Absorption curves of ruthenium complexes in aqueous solutions. Figure S3: 1H NMR spectra of the Ru complex. Figure S4: Mass spectra of the Ru complex. Figure S5: Quantitative analysis of the Ru complex in the Ru@MXene composite. Figure S6: The linear relationship between the optical power density (L) and the saturation temperature (T). Figure S7: Photographs of colonies of (a) *E. coli* and (b) *S. aureus* treated with the Ru@MXene composite at different concentration after light or non-light exposure. Figure S8: Relative viability of (a) *E. coli* and (b) *S. aureus* treated with the Ru@MXene composite at different concentration under light or dark conditions; and relative viability of (c) *E. coli* and (d) *S. aureus* treated with MXene, Ru and Ru@MXene under light or dark conditions with a xenon lamp (150 mW/cm^2^, 30 min) (mean ± SD, n = 3). Table S1: MBC of the Ru@MXene composite against *E. coli* and *S. aureus* after illumination. Table S2: Comparison of some nanomaterials with the photothermal effect for antibacterial properties. References [65,66,67,68,69,70] are cited in the Supplementary Materials.
