# Supplementary material for: Ru(II) Complex Grafted Ti3C2Tx MXene Nano Sheet with Photothermal/Photodynamic Synergistic Antibacterial Activity
Source: Nanomaterials (Basel). 2023 Mar 7;13(6):958. doi: 10.3390/nano13060958 (PMC10051588; doi:10.3390/nano13060958)
Supplement: Supplementary file 1 [file nanomaterials-13-00958-s001.zip › nanomaterials-2247791-supplementary.pdf]

nanomaterials

Supplementary Materials

Ru(II) Complex Grafted $Ti_3C_2T_x$ MXene Nano Sheet with Photothermal/Photodynamic Synergistic Antibacterial Activity

Xiaofang Liu [1], Hongchi Xie [1], Shi Zhuo [1], Yuanhong Zhou [1], Mohamed S. Selim [1,2], Xiang Chen [1,*] and Zhifeng Hao [1,*]

[1] Key Laboratory of Clean Chemistry Technology of Guangdong Regular Higher Education Institutions, School of Chemical Engineering and Light Industry, Guangdong University of Technology, Guangzhou 510006, P. R. China; fax6127@163.com (X.L.); hongchixie@163.com (H.X.); zhuos510@163.com (S.Z.); baobei2000818@163.com (Y.Z.); moh.selim_chem2006@yahoo.com (M.S.S.)

[2] Egyptian Petroleum Research Institute, Petroleum Application Department, Cairo Nasr city, Cairo 11727, Egypt; moh.selim_chem2006@yahoo.com (M.S.)

* Correspondence: chenxiang@gdut.edu.cn (X.C.); haozf@gdut.edu.cn (Z.H.)

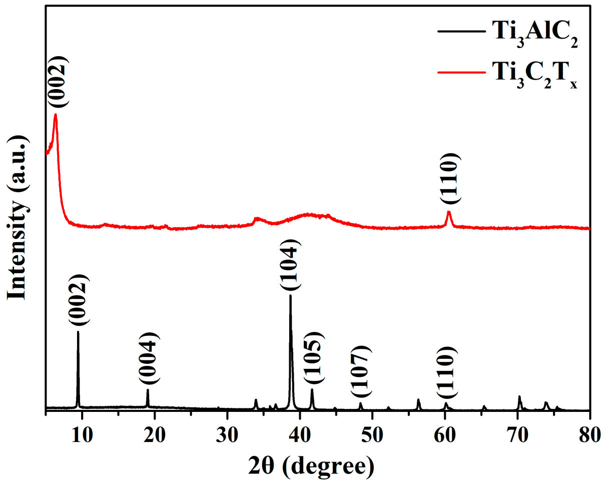

Figure S1. XRD spectra of Ti_3AlC_2 and $Ti_3C_2T_x$.

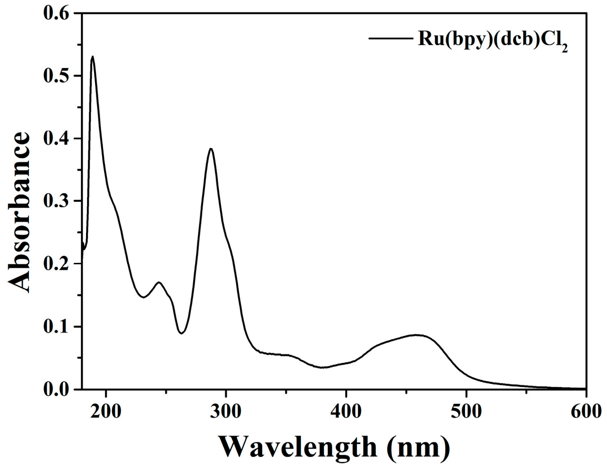

Figure S2. Absorption curves of ruthenium complexes in aqueous solutions.

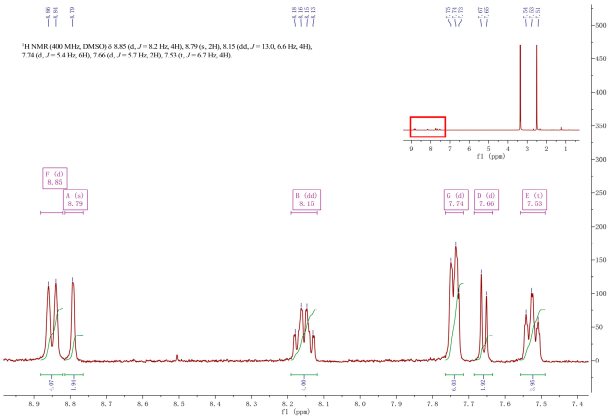

Figure S3. 1H NMR spectra of Ru complex.

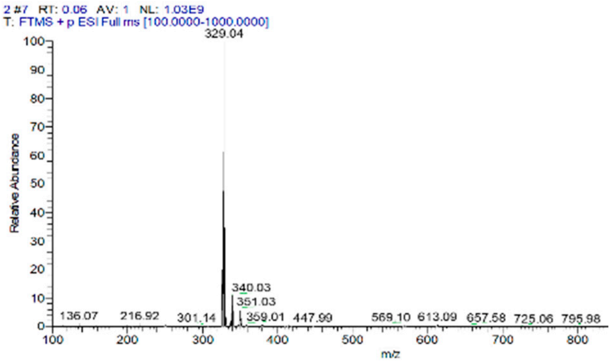

Figure S4. Mass spectra of Ru complex.

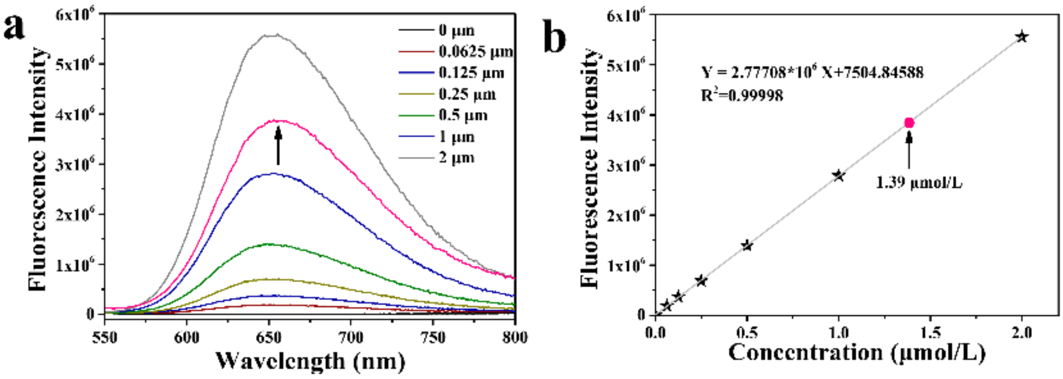

Figure S5. Quantitative analysis of Ru complex in the Ru@MXene composite. (**a**) The amount of Ru was determined by measuring the fluorescence intensity of Ru at 650 nm. From the fluorescence intensity of Ru@MXene (10 μg/mL), (**b**) the content of Ru in the Ru@MXene was quantified to be 1.39 μmol/L (10.13% (w/w)).

C_{Ru} = 16.7 nmol/mg × 101 g/mol × 100% = 0.17%
C_{Ru}=1.39 μmol/L × 728.65 g/mol=1.013 μg/mL
W%=1.013 μg/mL ÷ 10 μg/mL × 100% =10.13%.

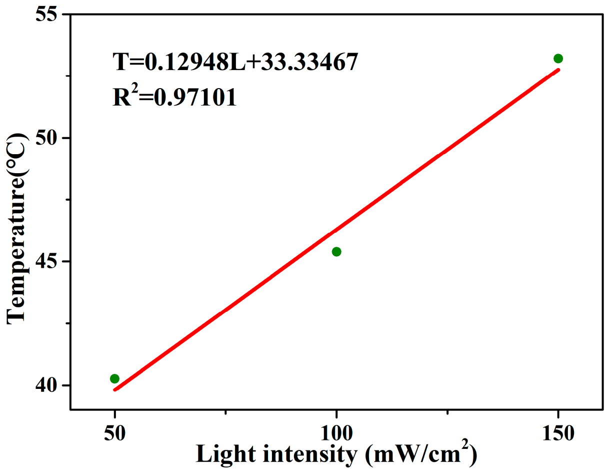

Figure S6. The linearly relationship between the optical power density (L) and the saturation temperature (T).

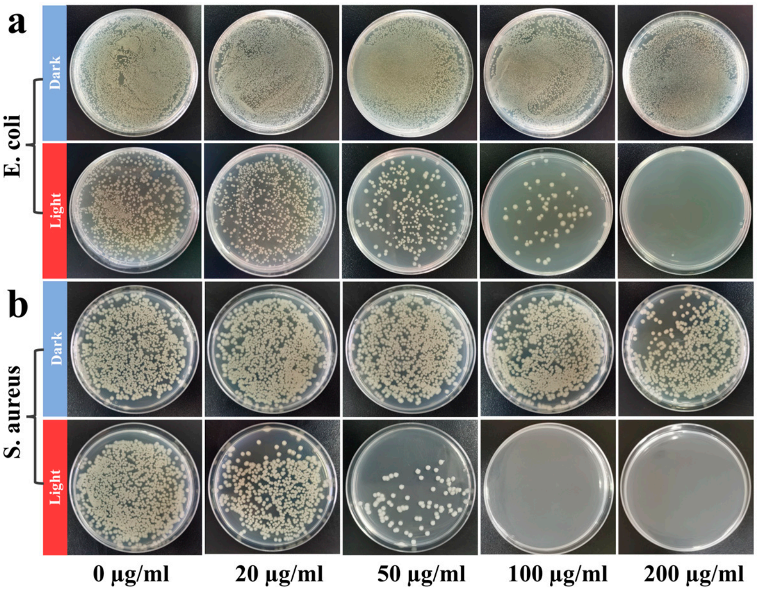

Figure S7. Photographs of colonies of (**a**) *E. coli* and (**b**) *S. aureus* treated with Ru@MXene composite at different concentration after light or non-light.

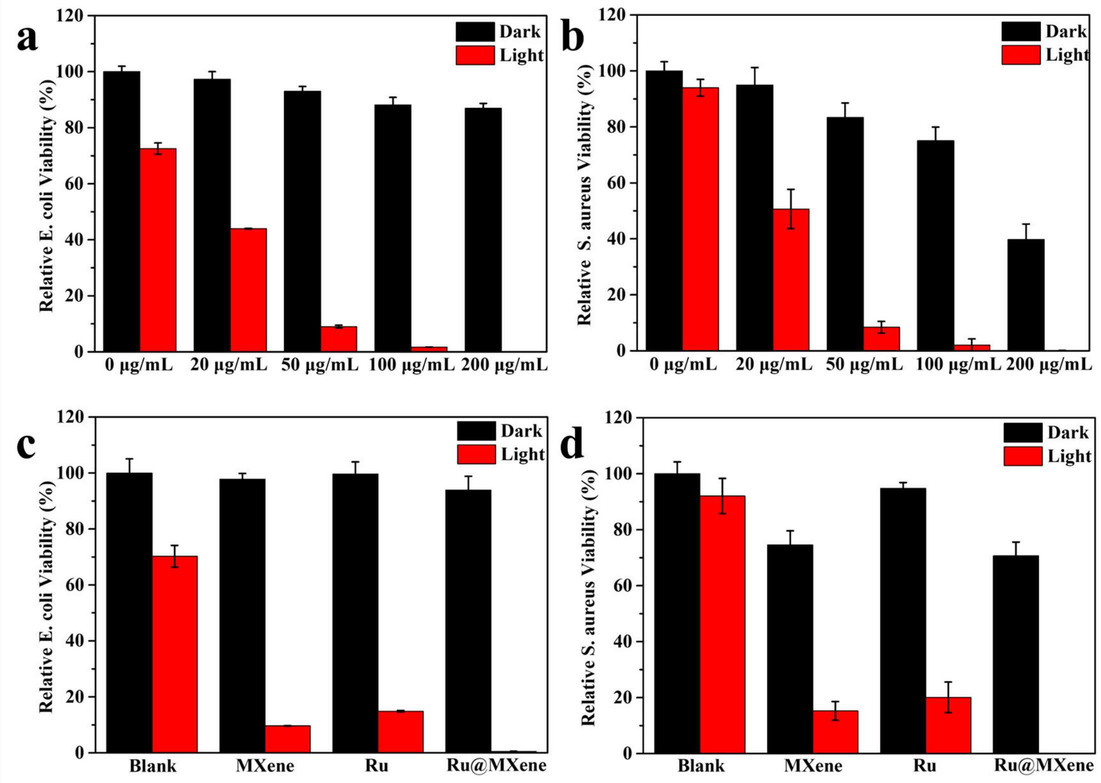

Figure S8. Relative viability of (**a**) *E. coli* and (**b**) *S. aureus* treated with Ru@MXene composite at different concentration under light or dark; and relative viability of (**c**) *E. coli* and (**d**) *S. aureus* treated with MXene, Ru and Ru@MXene under light or dark with xenon lamp (150 mW/cm^2, 30 min) (mean ± SD, n = 3). the colonies were counted and the viability was calculated using the following equation.

Relative viability % = Nm/Nc × 100%

where Nc is the number of bacteria in the control group, and Nm is the number of bacteria treated with different samples.

Table S1. MBC of Ru@MXene composite against *E. coli* and *S. aureus* after llumination.

**Bac-teria**	***E.coli***	***aureus***
MBC (μg/mL)	200	100

Table S2. Comparison of some nanomaterial with the photothermal effect for antibacterial.

**Nanomaterials**	**Antibacterial mechanism**	**Laser**	**Nanomaterial concentration**	**bacteria**	**Bacterial concentration**	**Antibacterial activity**	**Ref.**
Au@Bi_2S_3	PTT/PDT	808 nm, 1.5 W/cm^2, 3 min	*E. coli* (140 μg/mL), *S.aureus* (120 μg/mL)	*E. coli /S. aureus*	1 × 10^6 CFU/mL	~100%	1
MXene/ ZIF-8/PLA membrane	PTT/PDT	808 nm, 1 W/cm^2, 5 min	200 μg/mL	*E. coli/ MRSA*	1 × 10^6 CFU/mL	>99.0%	2
MXene/CoNWs coatings	PTT/PDT	808 nm, 1.5 W/cm^2, 20 min	-	*E. coli/ S. aureus*	1 × 10^6 CFU/mL	80.1% 92.74%	3

ZnTCPP/$Ti_3C_2T_X$	Photocatalytsis	visible light (λ>420 nm), 10min	200 μg/mL	*E. coli/ S. aureus*	1 × 10^6 CFU/mL	99.92% 99.86%	4
MOS_2/rGO(1%)	Nanozyme	xenon lamp 0.1 W/cm^2, 20min	50 μg/mL	*E. coli/ S. aureus*	5 × 10^4 CFU/mL	~100%	5
Ag-SMNPs@Cotton	PTT/ Ag^+	xenon lamp 0.7 W/cm^2, 10min	–	*E. coli/ S. aureus*	0.3 (OD)	~98.06% ~94.08%	6
GQDs-ε-PL/QCS/ 4 arm PEG-BA hydrogels	Chemotherapy/ PTT	xenon lamp 1 W/cm^2, 10min	–	*E. coli/ S. aureus/ P. aeruginosa*	1 × 10^6 CFU/mL	~96.6% ~97.1% ~98.0%	7
PSCN	Photocatalytsis	xenon lamp 0.25 W/cm^2, 2.5 h (S.aureus), 2 h (E.coli)	200 μg/mL	*E. coli/ S. aureus*	1 × 10^6 CFU/mL	97.0% 99.2%	8
BC/MoS_2-CS hydrogels	Membrane Disruption /PTT/PDT	xenon lamp 35 mW/cm^2, 30 min	MoS_2 (5 mg/mL)	*E. coli/ S. aureus*	10^8–10^9 CFU/mL	99.998% 99.988%	9
Ru@MXene	PTT/PDT	xenon lamp 0.15 W/cm^2, 30min	E. Coli (200 μg/mL), S.aureus (100 μg/mL)	*E. coli/ S. aureus*	1 × 10^7 CFU/mL	~100%	This work

References

1. Wang, W.-N.; Pei, P.; Chu, Z.-Y.; Chen, B.-J.; Qian, H.-S.; Zha, Z.-B.; Zhou, W.; Liu, T.; Shao, M.; Wang, H., Bi_2S_3 coated Au nanorods for enhanced photodynamic and photothermal antibacterial activities under NIR light. *Chem Eng J* **2020,** *397*, 125488.
2. Zhang, S.; Ye, J.; Liu, X.; Wang, Y.; Li, C.; Fang, J.; Chang, B.; Qi, Y.; Li, Y.; Ning, G., Titanium carbide/zeolite imidazole framework-8/polylactic acid electrospun membrane for near-infrared regulated photothermal/photodynamic therapy of drug-resistant bacterial infections. J Colloid Interface Sci **2021,** *599*, 390-403.
3. Liu, Y.; Tian, Y.; Han, Q.; Yin, J.; Zhang, J.; Yu, Y.; Yang, W.; Deng, Y., Synergism of 2D/1D MXene/cobalt nanowire heterojunctions for boosted photo-activated antibacterial application. *Chem Eng J* **2021,** *410*, 128209.
4. Cheng, H.; Wang, J.; Yang, Y.; Shi, H.; Shi, J.; Jiao, X.; Han, P.; Yao, X.; Chen, W.; Wei, X.; Chu, P. K.; Zhang, X., $Ti_3C_2T_x$ MXene Modified with ZnTCPP with Bacteria Capturing Capability and Enhanced Visible Light Photocatalytic Antibacterial Activity. *Sm*all **2022**, *18*, 2200857.
5. Wang, L.; Gao, F.; Wang, A.; Chen, X.; Li, H.; Zhang, X.; Zheng, H.; Ji, R.; Li, B.; Yu, X.; Liu, J.; Gu, Z.; Chen, F.; Chen, C., Defect-Rich Adhesive Molybdenum Disulfide/rGO Vertical Heterostructures with Enhanced Nanozyme Activity for Smart Bacterial Killing Application. Adv Mater **2020**, *32*, 2005423.
6. Liu, W.; Cheng, W.; Zhou, M.; Xu, B.; Wang, P.; Wang, Q.; Yu, Y., Construction of multifunctional UV-resistant, antibacterial and photothermal cotton fabric via silver/melanin-like nanoparticles. *Cellulose* **2022**, *29*, 7477–7494.
7. Cheng, C.; Zhong, H.; Zhang, Y.; Gao, X.; Wang, J.; Liu, J.; Han, X., Bacterial responsive hydrogels based on quaternized chitosan and GQDs-epsilon-PL for chemo-photothermal synergistic anti-infection in diabetic wounds. *Int J Biol Macromol* **2022**, *210*, 377-393.
8. Fang, Y.; Pei, S.; Zhuo, L.; Cheng, P.; Yuan, H.; Zhang, L., Phosphorus and sulfur codoped carbon nitride nanosheets with enhanced photocatalytic antibacterial activity and promotion of wound healing. Applied Surface Science **2022**, *586,* 152761.
9. Shen, H.; Jiang, C.; Li, W.; Wei, Q.; Ghiladi, R. A.; Wang, Q., Synergistic Photodynamic and Photothermal Antibacterial Activity of In Situ Grown Bacterial Cellulose/MoS_2-Chitosan Nanocomposite Materials with Visible Light Illumination. *ACS Appl Mater Interfaces* **2021**, *13*, 31193-31205.
